# Sub-CMC solubilization of dodecane by rhamnolipid in saturated porous media

**DOI:** 10.1038/srep33266

**Published:** 2016-09-13

**Authors:** Hua Zhong, Hui Zhang, Zhifeng Liu, Xin Yang, Mark L. Brusseau, Guangming Zeng

**Affiliations:** 1 College of Environmental Science and Engineering, Hunan University, Changsha 410082, China; 2 School of Water Resources and Hydropower Engineering, Wuhan University, Wuhan 430070, China; 3 Department of Soil, Water and Environmental Science, University of Arizona, Tucson, Arizona 85721, USA; 4 Key Laboratory of Environmental Biology and Pollution Control (Hunan University), Ministry of Education, Changsha, 410082, China

## Abstract

Experiments were conducted with a two-dimensional flow cell to examine the effect of monorhamnolipid surfactant at sub-CMC concentrations on solubilization of dodecane in porous media under dynamic flow conditions. Quartz sand was used as the porous medium and artificial groundwater was used as the background solution. The effectiveness of the monorhamnolipid was compared to that of SDBS, Triton X-100, and ethanol. The results demonstrated the enhancement of dodecane solubility by monorhamnolipid surfactant at concentrations lower than CMC. The concentrations (50–210 μM) are sufficiently low that they do not cause mobilization of the dodecane. Retention of rhamnolipid in the porous medium and detection of nano-size aggregates in the effluent show that the solubilization is based on a sub-CMC aggregate-formation mechanism, which is significantly stronger than the solubilization caused by the co-solvent effect. The rhamnolipid biosurfactant is more efficient for the solubilization compared to the synthetic surfactants. These results indicate a strategy of employing low concentrations of rhamnolipid for surfactant-enhanced aquifer remediation (SEAR), which may overcome the drawbacks of using surfactants at hyper-CMC concentrations.

Soil and groundwater contamination by hydrophobic organic compounds (HOCs) represents a severe environmental problem due to the widespread use, storage, and disposal of organic solvents, fuels, and petroleum products. The contamination is difficult to remediate due to the hydrophobic nature of these organic compounds, as indicated by low water solubility and strong tendency to partition into organic phases^1^,^2^. Once released in liquid form, they tend to migrate into the subsurface and form non-aqueous phase liquids (NAPLs)^3^. NAPLs are often present as capillary trapped ganglia in pore space in saturated zones that formed during the migration downward under gravity (dense-NAPLs)^4^,^5^, or through fluctuation of the water table (light-NAPLs)^6^,^7^. Entrapped NAPLs dissolve slowly into groundwater, which causes long-term groundwater contamination.

Due to low water solubility of the HOCs, conventional pump-and-treat technologies have been proven ineffective in treating source zones containing NAPLs, e.g., decades or even longer may be required to remove the contaminants completely^8^,^9^. Therefore, surfactant-enhanced aquifer remediation (SEAR) and other enhanced solubilization methods have been developed to accelerate contaminant-mass removal for such systems^10^,^11^,^12^. SEAR consists of two primary removal mechanisms, which are (1) mobilization of trapped NAPLs caused by interfacial tension reduction, and (2) micellar solubilization^13^,^14^. Due to potential issues with fugitive NAPL migration associated with mobilization^11^,^15^,^16^, solubilization-based SEAR has gained more attention.

Based on the conventional micelle-based mechanism for surfactants to enhance solublization of HOCs, significant solubilization only occurs at surfactant concentrations higher than the critical micelle concentration (CMC), at which micelles form in significant quantities^3^,^15^,^17^. As a result, almost all of the prior studies focused on NAPL removal were conducted at standard hyper-CMC surfactant concentrations (usually much higher than the CMCs). For example, various types of surfactants for solubilizing chlorinated solvents at hyper-CMC concentrations were reported^13^. The removal of NAPLs by hyper-CMC surfactants from porous media has also been investigated under dynamic flow conditions^18^,^19^. Two major concerns toward such standard use of high concentrations of surfactant are (1) introduction of large amounts of surfactant into the aquifer, which may cause secondary contamination or other impacts to the system, and (2) potential impact on migration and distribution of NAPLs via mobilization. These drawbacks have significantly limited the application of SEAR technology.

The results of recent studies, however, have shown that substantial solubilization may also occur at surfactants concentrations below the CMCs^20^,^21^. Specifically, it was observed that rhamnolipid biosurfactant exhibited strong alkanes-solubilization activity at concentrations significantly lower than CMC through an aggregate-formation mechanism^22^. In addition, synthetic surfactants, SDBS and TX-100, were shown to enhance the solubility of hexadecane at sub-CMC concentrations^23^. It is anticipated that enhanced solubilization using sub-CMC concentrations may not have the drawbacks of the standard solubilization approach employing high-concentrations of surfactants. The prior studies, however, were implemented exclusively in batch-reactor systems, which are not sufficient to test solubilization behavior under dynamic flow conditions.

The objective of this study was to examine the feasibility of using sub-CMC surfactant concentrations for solubilization of NAPL in porous media under dynamic flow conditions, so as to overcome the drawbacks of the standard hyper-CMC SEAR method. A two-dimensional flow cell was used for the experiments. Quartz sand was used as the porous medium. *n-*Dodecane, which shows great solubility in the presence of sub-CMC rhamnolipid in batch experiments^22^, was used as the immiscible liquid. Monorhamnolipid, SDBS, and Triton X-100 were selected as the surfactants, and ethanol as a co-solvent was also used for comparison.

## Results and Discussion

### Batch solubilization of dodecane by surfactants under static conditions

The results of solubilization of dodecane under static condition in batch experiment are shown in Fig. S1a,SI. The dodecane concentration in AGW is below the detection limit of the GC-FID (0.1 mg/L) during the entire period of the test, which is consistent with the low water solubility of dodecane at this temperature (0.02 μM or 0.003 mg/L). For all the surfactant treatments, the concentrations of dodecane become stable or show significantly lowered increasing rate after 48 hours, indicating solubilization equilibrium. The presence of surfactants with concentrations below or equal to CMC moderately increases the solubility of dodecane (45 to 110 μM, or 8 to 19 mg/L). The solubility is increased significantly by the rhamnolipid at a concentration of 1050 μM (5 × CMC) as would be anticipated due to formation of large quantities of micelles in the aqueous phase.

As shown in Fig. S1a,SI, solubilization of dodecane by surfactants under static condition is a rate-limited process and the solubility of dodecane at 24 h is 10 and 20 μM for 50 and 210 μM of rhamnolipid, respectively. In contrast, the solubility of dodecane at 24 h in the presence of 50 and 210 μM of rhamnolipid under agitation conditions are 224 and 826 μM, respectively,which are similar to that of our prior study employing sub-CMC rhamnolipid for alkane solubilization^22^ and are significant higher than those obtained under static conditions. Such a difference indicates the importance of relative movement between water and dodecane phases on aggregate-based sub-CMC solubilization of the dodecane.

The concentration of surfactant versus time for static solubilization of dodecane is shown in Fig. S1b,SI. The concentrations of surfactants remain unchanged within 144 h, indicating no partition of surfactants into dodecane. This is consistent with the general assumption that the ionic surfactants and most nonionic surfactants have minimal partition to hydrophobic organic phases^24^,^25^.

### Observation of aggregates for solubilization of dodecane

**Figure 1:**
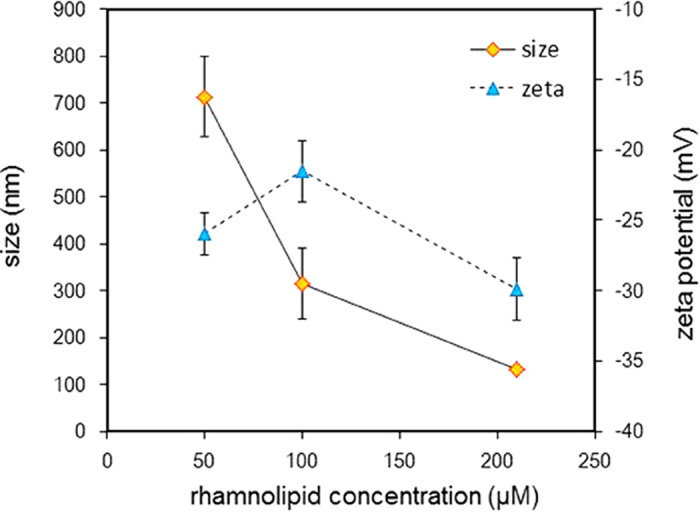
The DLS size and zeta potential of dodecane-monoRL aggregates in the effluent of the flow cell in solubilization test. Samples were collected at the plateau of dodecane elution curves (2.5~4.5 PV).

Effluent samples collected during the flow-cell experiments were analyzed for the presence of aggregates using dynamic light scattering (DLS). Typical number-based particle size distribution profiles are presented in Fig. S2,SI. Only one peak was observed for the profiles of all rhamnolipid concentrations, indicating one size of aggregate. As shown in Fig. 1, the size of dodecane-rhamnolipid aggregates decreased with the increase of rhamnolipid concentration. This result is similar the results of our prior batch study, which shows that dodecane-monoRL aggregate size decreases with the increase of rhamnolipid concentration at rhamnolipid concentrations below CMC due to increase of monoRL access at the aggregate surface and thus curvature of the surface^22^,^23^. Zeta potentials of the aggregates are between −20 and −30 mV with a peak at the rhamnolipid concentration of 100 μM. The result shows that the aggregates are negatively charged, which is due to dissociation of the carboxyl groups in monoRL molecules at the aggregate surface. This result, however, is different from the results of our prior batch experiments on dodecane solubilization by rhamnolipid, in which the aggregate size decreased monotonously with increase of rhamnolipid concentration at sub-CMC concentrations in a phosphate buffer. In this study, the background AGW has limited buffer capacity and the pH of monoRL solution decreases with increasing concentration of monoRL concentration (6.7, 6.5 and 6.0 for monoRL concentration of 50, 100, and 210 μM, respectively).The degree of dissociation for monoRL molecules (carboxyl group) decreases with the decreasing pH. Increasing monoRL concentration causes increase of monoRL access at the aggregate surface which contributes to a more negatively charged surface, and decrease of monoRL dissociation which tends to cause a less negatively charged surface. The peak of zeta potential at 100 μM of monoRL may be a result of counteract of these two effects.

**Figure 2:**
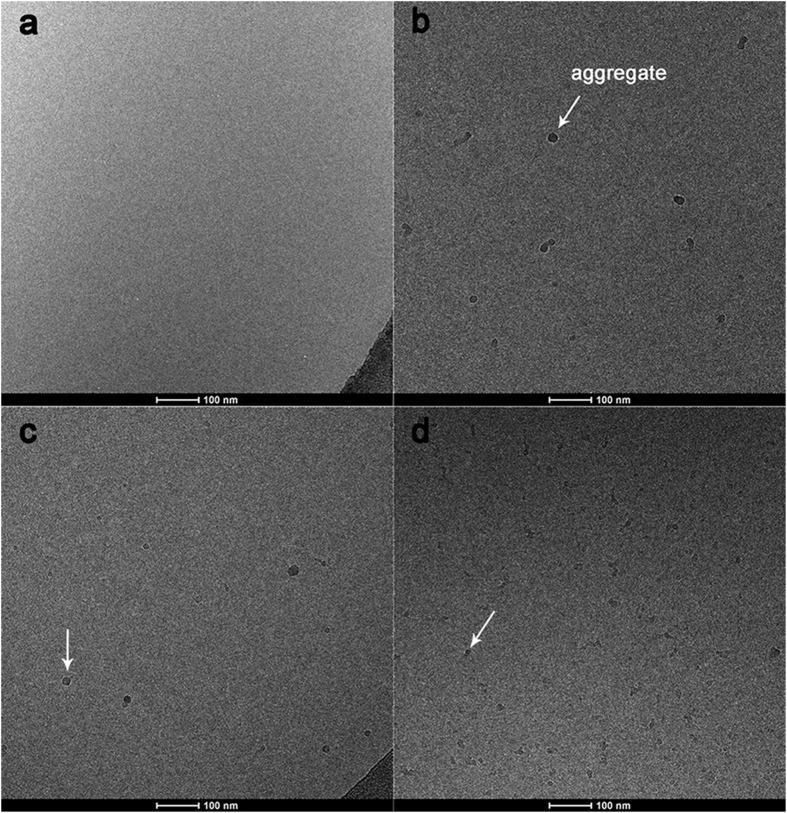
Representative cryo-TEM micrographs of dodecane-monoRL aggregates in the effluent of the flow cell in solubilization test with rhamnolipid concentrations of (**a**) 0 μM, (**b**) 50 μM, (**c**) 100 μM, and (**d**) 210 μM in influent. Samples were collected at the plateau of dodecane elution curves (2.5~4.5 PV).

Typical cryo-TEM images of the same effluent samples used in DLS aggregate size measurements are presented in Fig. 2 (the images used for measuring the cryo-TEM-based size distribution for the aggregates are presented in Fig. S3,SI). No aggregates were observed in the effluent samples during injection of AGW (Fig. 2a). In contrast, spherical aggregates with diameter less than 100 nm are observed with injection of sub-CMC rhamnolipid solutions (Fig. 2b–d). The results of the DLS-based and cryo-TEM-based aggregate size distribution are presented in Fig. S4,SI. The natural logarithm of aggregate sizes, obtained with either DLS or cryo-TEM method, follows Gaussian distribution. The mean sizes calculated from Gaussian regression are presented in Table 1. The DLS-based size is much larger than the cryo-TEM-based size. This is because DLS measures the hydrodynamic size of particles, which is larger than the physical size^26^. The cryo-TEM aggregate size decreased with the increase of rhamnolipid concentration, which is the same trend as observed for the DLS data.

### Sub-CMC solubilization of dodecane by surfactants

**Figure 3:**
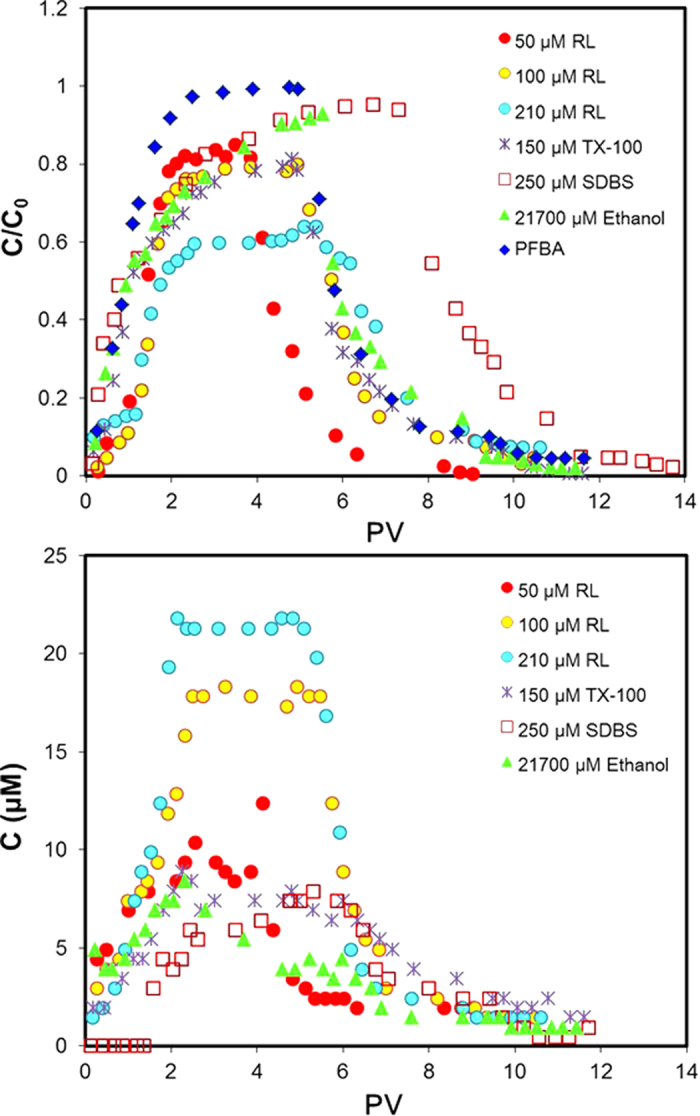
Breakthrough curves of the surfactants and ethanol (**a**) and elution curves of dodecane (**b**) for dodecane solubilization in the 2-D flow cell. Dodecane saturation (*S*_n_) is 0.0035. Area containing dodecane is 136 cm^2^. Pore water velocity is 0.2 cm/min. Temperature is 25 °C.

The breakthrough curves (BTCs) of the PFBA, surfactants and ethanol are shown in Fig. 3a. The PFBA BTC shows slight shouldering and tailing, which is due to inuniformity of the flow field associated with local heterogeneity of the porous medium caused by the trapped dodecane in the center of the flow cell. However, the BTC shows no significant retardation or retention, which is a typical feature for nonreactive tracers. The ethanol BTC exhibits a limited degree of retardation. This could be due one or more of the following three mechanisms (in order of anticipated likelihood), bulk partitioning to the NAPL, solid-phase adsorption, and partitioning to the NAPL-water interface.

Measurable retardation is observed for rhamnolipid, which is in contrast to the synthetic surfactants and ethanol, indicating strong activity of the rhamnolipid to partition to interfaces. Compared to the PFBA BTC, all of the surfactant BTCs exhibit the existence of steady concentration plateaus at values less than C/C_0_ = 1. In addition, mass-balance calculations show that surfactant recoveries are significantly lower than mass inputs (77%, 79%, and 69% for rhamnolipid concentrations of 50, 100, and 210 μM, respectively, 84% for TX-100, and 95% for SDBS). This is indicative that surfactant transport is influenced by a retention process that is effectively irreversible. The magnitude of retention ranges from ~5% (250 μM of SDBS) to ~40% (210 μM of rhamnolipid). These result are very different from our prior observations that monoRL did not show retention during transport in saturated sandy soils containing no NAPL^27^. Given the observed presence of surfactant aggregates in the flow-cell effluent, the apparent irreversible-retention behavior of the surfactants observed for the flow-cell experiments is attributed to the presence of the aggregates, whose transport would be governed by colloid transport mechanisms.

The aggregate size is smaller than 500 nm based on the result of DLS size measurement, producing a particle-to-medium size ratio of ~0.001, which is much smaller than the empirical threshold (0.05) for significant straining effects to occur^28^. As a result, straining is not the dominant mechanism for the retention. Because both the aggregate and the medium surfaces are negatively charged (shown by zeta potentials), the attachment of the aggregates is occurring under unfavorable conditions. Given that the sand is geochemically homogeneous, the mechanisms most likely contributing to aggregate retention are adsorption at secondary minima and at local physical heterogeneities (crevices, pits, etc).

The elution curves of dodecane in the solubilization tests are presented in Fig. 3b. The highest dodecane mass discharge obtained from the curves is 21 mg for 210 μM of rhamnolipid, which is significantly lower than the total dodecane mass (~4 g), showing that the solubilization within the time frame of the experiment did not cause significant change of the dodecane configuration (and that the configuration is essentially the same for all of the tests). The AGW caused minimal solubilization (dodecane concentration was below detection limit, data not shown). Enhanced solubilization of dodecane by the rhamnolipid is observed at all the rhamnolipid concentrations tested (50, 100, and 210 μM), and the dodecane concentration in the effluent presented by the plateau of the elution curves is greater for higher rhamnolipid concentration. These dodecane concentrations, however, is significantly lower than that caused by hyper-CMC rhamnolipid (1050 μM), which is approximately 500 μM (Fig. S5,SI, 4 to 8 PV). The results show that hyper-CMC rhamnolipid has the advantage to cause solubilization, probably due to presence of large quantity of micelles. 150 μM of Triton X-100 and 250 μM of SDBS also enhanced solubilization of dodecane indicated by the elevated plateau of the elution curves, however, both of the plateaus are lower than that for 50 μM of rhamnolipid. Ethanol as a co-solvent at a concentration significantly higher than that of the surfactants (21700 μM) caused limited dodecane solubilization with a plateau lower than that for the surfactants. The plateau dodecane concentrations for the surfactants are smaller than the equilibrium concentrations in batch static solubilization experiment, due to the low contact time (hydraulic residence time in the dodecane zone of 1.4 h versus contact time of 96 h in batch experiment), dilution effects caused by the absence of NAPL in the top and bottom regions of the flow cell, and retention of the dodecane-rhamnolipid aggregates in the medium. However, these plateau dodecane concentrations under dynamic flow condition are similar to or higher than dodecane concentration at 24 h under static condition (Fig. S1a,SI). These results also indicate the importance of relative movement between water and dodecane phases on formation of dodecane-surfactant aggregate, which is probably due to the enhanced diffusion of aggregates from the interface to bulk aqueous phase.

Because the dodecane concentration produced from solubilization at the source zone is not available, the exact retention ratio of the aggregates is unknown. The magnitude of the retention, however, can be estimated using the retention ratio of rhamnolipid. In our prior batch studies testing solubilization of alkanes by monoRL in a salt medium, 23% of the monoRL molecules were associated with the aggregates at sub-CMC concentrations and the other 77% were freely dissolved in bulk solution, based on surfactant partition theories and mass balance calculation^25^,^29^. In this study the retention ratios of monoRL at a *C*_0_ of 50 μM and 100 μM are 18% and 20%, respectively. By assuming that percentage of monoRL associated with the aggregate for the solubilization is 23% (could be lower due to the lower salt concentration in background solution in this study) and retention of the monoRL is caused only by retention of aggregates, the retention rate of the aggregates may be higher than 80%. Such estimation implies significant retention of the dodecane-monoRL aggregates in the porous medium. Such retention of NAPL-monoRL aggregates, however, is not a problem for application of this method because it is a dispersion of NAPL in the treated zone that can be controlled. In addition, such dispersion can enhance the bioavailability of the contaminants and thus biodegradation of the contaminants (rhamnolipid is a biosurfactant that is readily degradable). The large specific surface area of the aggregates is also beneficial to mass transfer of the contaminants. A simple bioremediation or solvent extraction technique can be used to remove the trapped aggregates, if necessary.

## Conclusions

The results of this study demonstrate the enhancement of dodecane solubility in quartz sand medium under 2-D flow condition by monorhamnolipid surfactant at sub-CMC concentrations, and the solubilization occurs at rhamnolipid concentrations sufficiently low to not cause mobilization of the NAPL. The solubilization is based on aggregates formation mechanism and the aggregate size decreases with increasing rhamnolipid concentration. This provides an alternative method for implementation of SEAR technology for remediation of NAPL contaminated sites. Future studies will be focused on testing the solubilization in a broad range of porous media and the effect of flow conditions on the solubilization, and the time-dependent features of the solubilization as well.

## Experimental Materials and Methods

### Materials

The monorhamnolipid (monoRL, purity > 99%) was purchased from Zijin Biological Technology Co., Ltd. (Zhejiang, China). The methods and results for component analysis were described by Zhong *et al.*^30^. SDBS, Triton X-100 (TX-100) and perfluorobenzoic acid (PFBA) were purchased from Sigma-Aldrich (St Louis, Mo., U.S.). HPLC grade ethanol was purchased from Sinopharm Chemical Reagent Co., Ltd. All the surfactant and ethanol solutions were prepared with artificial ground water (AGW) as the background solution (ingredients per liter: 0.006 g NaCl, 0.012 g CaSO_4_, 0.012 g NaHCO_3_, 0.002 g KNO_3_, 0.035 g MgSO_4_·7H_2_O, and pH 6.8)^29^. As shown in Fig. S6,SI, the CMCs of the SDBS, TX-100 and monoRL in AGW are 584, 302, and 210 μM, respectively, determined using interfacial–tension measurement method (see supporting information for details of the methods and results).

*n*-Dodecane (purity ≥ 99%) was obtained from Sigma-Aldrich (St. Louis, Mo., U.S.). For visualization purpose, Oil-Red-O (Shifeng Biological Technology Co., Ltd. Shanghai, China), an organic soluble dye, was used at a concentration of 4 × 10^−4 ^M to colorize dodecane. Previous studies have shown that the addition of Oil-Red-O at concentrations lower than 10^−3 ^M does not affect the interfacial properties of n-dodecane^31^. All other chemicals were of analytical grade. Ultra-pure water was produced by UPT-II-40 (Ulupure, Chengdu, China) with an electrical resistivity of 18.3 mΩ∙cm. The molecular structures of the surfactants, PFBA and ethanol are presented in Table S1,SI.

The quartz sand was obtained from Zhilei Construction Co. Ltd (Guangzhou, China). The sand was ultrasonically cleaned, air-dried and then screened. The segment with the size of 26–35 mesh (0.50–0.78 mm) was used as the porous medium for the experiments. The major constituents of the sand are SiO_2_ (≥99.0%), Al_2_O_3_ (≤0.5%), and Fe_2_O_3_ (≤0.15%). Organic matter content was 0.3%, which is measured using the combustion-mass loss method. The zeta potential of the sand in AGW was −30 mV, which measured using a ZEN3600 Zetasizer Nano (Malvern Instruments, Malvern, UK) after the sand was ground into powder.

### Batch static solubilization experiment

Solubilization of dodecane by surfactants (monoRL, SDBS and TX-100) under static conditions was examined using batch experiments. Briefly, 20 mL of surfactant solution with designated concentrations were added to 30-mL conical flasks. Then 100 μL of dodecane was added very slowly to the top of the solution using a pipette with the tip almost contacting the solution surface. By this means the dodecane liquid did not go into the solution but spread on the surface of the solution. These flasks were then carefully placed in an incubator with minimal disturbance of the inside solution. At predetermined time points, 4 mL aliquots of the solution were sampled slowly from the bottom of flask using a pipette. The method of processing these aliquots of samples to obtain subsamples completely free of undissolved hexadecane was described by Zhong *et al.*^32^. Some of the subsamples were then used for dodecane concentration measurement by gas chromatography (Agilent GC 6890N). The surfactant stock solutions and the 144 h samples were also analyzed for surfactant concentrations.

For some rhamnolipid concentrations, test on solubilization of dodecane by the monorhamnolipid under shaking conditions was also conducted. Briefly, 50 μL of dodecane was pipetted and spread on the bottom of a 30-mL conical flask. 10 mL of mono-RL solution was added to the flask and incubated on the reciprocal shaker at 30 °C, 120 rpm for 24 h. Then the solutions were allowed to stand still for 2 h for phase separation. The methods for sample collection, processing, and dodecane concentration analysis were described by Zhong *et al.*^22^,^32^.

### Solubilization experiments with the 2-D Flow cell

A schematic diagram and a photographic image of the flow cell system are shown in Fig. S7,SI. The flow cell consists of six glass plates (1.2 cm in thickness), five of which were glued together using UV glue. The dimension of the inside space for porous medium was 57 cm (length) × 50 cm (height) × 1.2 cm (thickness). Two stainless-steel tubes with square cross-section (1 cm × 1 cm × 50 cm) at the left and right sides of the medium space were used as the flow distributors for influent and effluent, respectively. Before installing into the flow cell, the two tubes were cut open (1 cm × 40 cm) at the side facing porous medium. 100-mesh stainless steel wiring nets were used to wrap around the tubes to retain the porous medium and promote uniform flow along the vertical interfaces at each side of the porous medium. Small round tubes were welded onto the center of the top end of stainless-steel tubes and used to connect the influent and effluent tubings.

The 26–35 mesh quartz sand was packed into the flow cell using a water-saturated packing method, for which the water level was always maintained 5 cm above the sand during packing. When the height of sand reached 42 cm, the water on top of the sand was removed and 5 ml (or 3.7 g) of dodecane dyed with Oil-Red-O was injected into the 2-D cell using a 5-mL syringe equipped with a 40 cm stainless steel needle. The injection point was approximately 14 cm from the bottom of the sand and 28.5 cm from either side. The injected dodecane was allowed to redistribute in the next 24 h. Then the sixth plate (57 × 10 cm × 1.2 cm) was inserted until it had full contact with the top of the sand. Glass cement was used to seal the top plate to the flow cell. De-aired AGW was injected for the next 3 days. No air bubbles were detected in the flow cell thereafter, which ensured a fully saturated condition for the following experiments. The total pore volume of the packed flow cell was 2825 cm^3^, with a bulk density of 1.6 g/cm^3^ and a porosity of 0.42.

Initial experiments were conducted to examine the potential for the enhanced-solubilization solutions to cause NAPL mobilization. This was done by consecutive injections of rhamonolipid solutions at concentrations of 0 (AGW), 50, 210, and 1050 μM. The solutions were introduced by a valveless piston pump (QSY, Fluid Metering INC, USA) at a flow rate of 4 ml/min (Darcy velocity of 0.083 cm/min, or pore water velocity 0.2 cm/min), and then eluted with AGW. For each rhamnolipid concentration, the effluent was collected and the dodecane concentration was measured for calculation of mass discharge. A camera (Canon EOS 7D) was fixed on a tripod 1 m from the 2-D cell. Dodecane NAPL morphology was photographed at designated time points during the injections. The images were processed using Photoshop software to enhance contrast. The results showed no observable changes in NAPL distribution for the first three injections. A modest increase in NAPL-resident area was observed after injection of the 1050 μM rhamnolipid solution (Fig. S8,SI). Inspection of the effluent samples revealed the absence of emulsions or separate dodecane blobs for all injections. These results indicate that the 50 and 210 uM RL solutions did not cause mobilization of the dodecane.

Loss of dodecane from the flow cell during the initial experiments was 18% calculated from the mass discharge, with the vast majority associated with the 1050 μM flood (elution curve of dodecane and breakthtough curve of rhamnolipid for injection of 1050 μM rhamnolipid is shown in Fig. S5,SI. Mobilization of the dodecane stopped at ~4 PV and dodecane in the effluent between ~4 and ~8 PV was from solubilization of the trapped dodecane). The local residual NAPL saturation (*S*_nlr_) is estimated to be 0.04 in the NAPL-resident zone (17 cm × 12 cm area). The apparent, flow-cell-wide NAPL saturation (*S*_n_) is 0.0035.

For the solubilization experiments, predetermined concentrations of PFBA, surfactants (monoRL, SDBS or TX-100), or ethanol solutions were injected into the flow cell at a flow rate of 4 ml/min and then followed by elution of AGW. At predetermined time intervals, effluent samples were collected using 10-ml borosilicate glass vials with screw caps. The concentration of injected chemicals and dodecane in the samples were measured. For some samples collected at 2.5~4.5 PV with rhamnolipid injection, the dynamic light scattering size (DLS size) and zeta potential of particles were also measured, and the morphology of particles was observed using Cryo-Transmission Electron Microscopy (cryo-TEM). Between experiments, the flow cell was flushed with AGW for at least 6 PVs. No mobilization of the dodecane phase was observed during the entire solubilization experiment.

### Analytical Methods

Dodecane and ethanol concentrations in the samples were analyzed using an Agilent 6890N gas chromatograph (GC) equipped with a flame ionization detector (FID). The columns for dodecane and ethanol analysis were HP-5 (30 m × 0.32 mm × 0.25 μm) and HP-INNOWAX (30 m × 0.32 mm × 0.25 μm), respectively. The details for sample processing and GC conditions for dodecane analysis were described by Zhong *et al.*^32^. For ethanol measurement, the aqueous samples were injected directly. Helium was used as the carrier gas. The injection volume was 1 μL, with a split injection ratio of 70:1. The temperatures of the injection port and the detector are 280 °C and 290 °C, respectively. The oven temperature ramped from 50 to 120 °C at a rate of 20 °C/min with no holds.

SDBS and TX-100 concentrations in the samples were measured using an ultraviolet spectrophotometric method described by Zeng *et al.*^33^. The measurement of monorhamnolipoid was based on phenol-sulfuric acid method described by Zhong *et al.*^34^.

The DLS size and the zeta potential of aggregates in the effluent samples were measured using ZEN3600 Zetasizer Nano (Malvern Instruments, U.K.). The details of the methods were described in our previous studies^22^,^23^,^35^. pH of these effluent samples were also measured using a pH meter.

The aggregate morphology of was observed using Cryo-Transmission Electron Microscopy (cryo-TEM) and the method details were described by Zhong *et al.*^23^. Nano measurer 1.2.5 software (Shanghai, Fudan University) was utilized to process the micrograph images for particle size. Before the particles in the images were circled, screen ruler was calibrated by the reference bar in the image. Then the diameter of every circle was measured by pairing to the screen ruler. After all the circles of the image were depicted, a table of particle sizes statistics was obtained. In order to obtain statistically representative data of aggregate size distribution, the size data were collected on 100~200 particles from multiple images for each rhamnolipid concentration tested.

## Additional Information

**How to cite this article**: Zhong, H. *et al.* Sub-CMC solubilization of dodecane by rhamnolipid in saturated porous media. *Sci. Rep.* **6**, 33266; doi: 10.1038/srep33266 (2016).

## Supplementary Material

Supplementary Information

## Figures and Tables

**Table 1:** Parameters of Gaussian regression for delineating distribution of dodecane-monoRL aggregate size obtained using DLS and cryo-TEM methods.

Mono-RL	DLS				cryo-TEM			
	μ^a^	σ^2^^b^	R^2^	*d* (nm)^c^	μ	σ^2^	R^2^	*d* (nm)
50 μM	6.04	0.21	0.99	**420**	2.91	0.32	0.96	**18**.**4**
100 μM	4.99	0.22	0.97	**147**	2.58	0.28	0.97	**13**.**2**
210 μM	4.61	0.23	0.99	**101**	2.21	0.23	0.97	**9**.**1**

^a^Mean of ln*d* obtained from Gaussian regression.

^b^Variance of ln*d* obtained from Gaussian regression.

^c^The mean aggregate size obtained using *d* = e^μ^.
