# Supplementary material for: Sub-CMC solubilization of dodecane by rhamnolipid in saturated porous media
Source: Sci Rep. 2016 Sep 13;6:33266. doi: 10.1038/srep33266 (PMC5020404; doi:10.1038/srep33266)
Supplement: Supplementary Information [file srep33266-s1.pdf]

Supporting Information

Sub-CMC solubilization of dodecane by rhamnolipid in saturated porous media

Hua Zhong[1, 2, 3] *, Hui Zhang[1, 2], Zhifeng Liu[1,2] *, Xin Yang [1, 2], Mark L Brusseau[3], Guangming Zeng[1,2]

[1]College of Environmental Science and Engineering, Hunan University, Changsha 410082, China;

[2] Key Laboratory of Environmental Biology and Pollution Control (Hunan University), Ministry of Education, Changsha, 410082, China;

[3] Department of Soil, Water and Environmental Science, University of Arizona, Tucson, Arizona 85721;

Corresponding author:

E-mail: zhonghua@email.arizona.edu; Mailing address: Department of Soil, Water and Environmental Science, University of Arizona, Tucson, Arizona 85721; Tel.: +1-520-626-4191; Fax: +86-731-88823701

E-mail: lzf18182002@163.com; Mailing address: College of Environmental Science and Engineering, Hunan University, Changsha 410082, China; Tel.: +86-133 4869 8016; Fax: +86-731-88823701

Table S1 The molecular weight and structure of chemicals used in this study

Chemical name	mol wt (g/mol)	molecule structure
Monorhamonolipid	504[a]	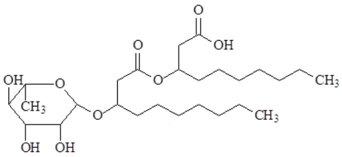 [b]
SDBS	348	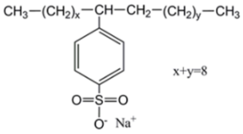
TX-100	647	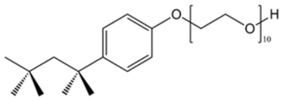
PFBA	212	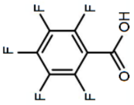
Ethanol	46	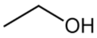
Dodecane	170	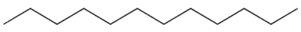

[a] Average molecular weight

[b] Molecular structure of the major species

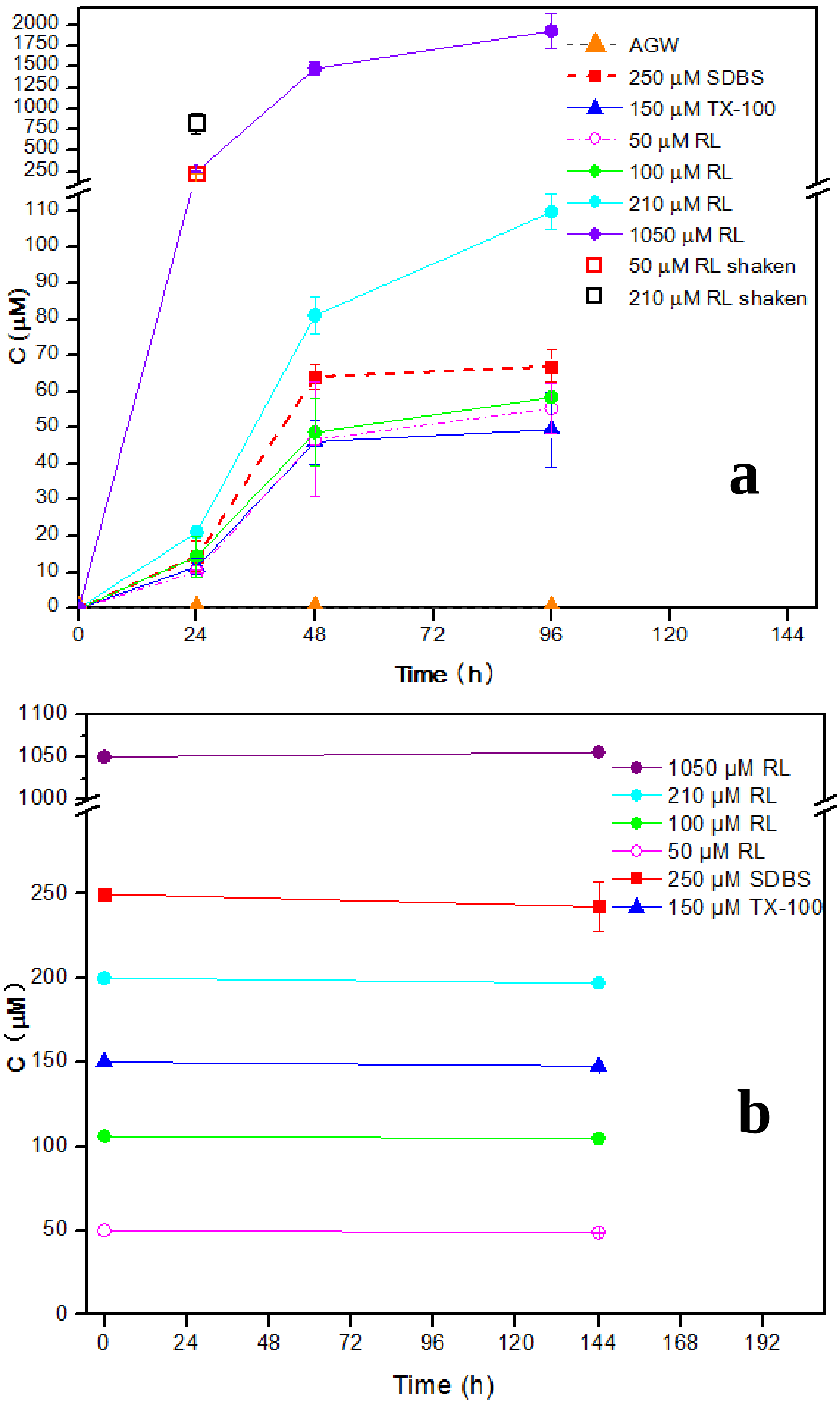

Figure S1 Concentrations of dodecane (a) and surfactants (b) versus time in aqueous phase in batch static solubilization experiments. Temperature is 25 ℃.

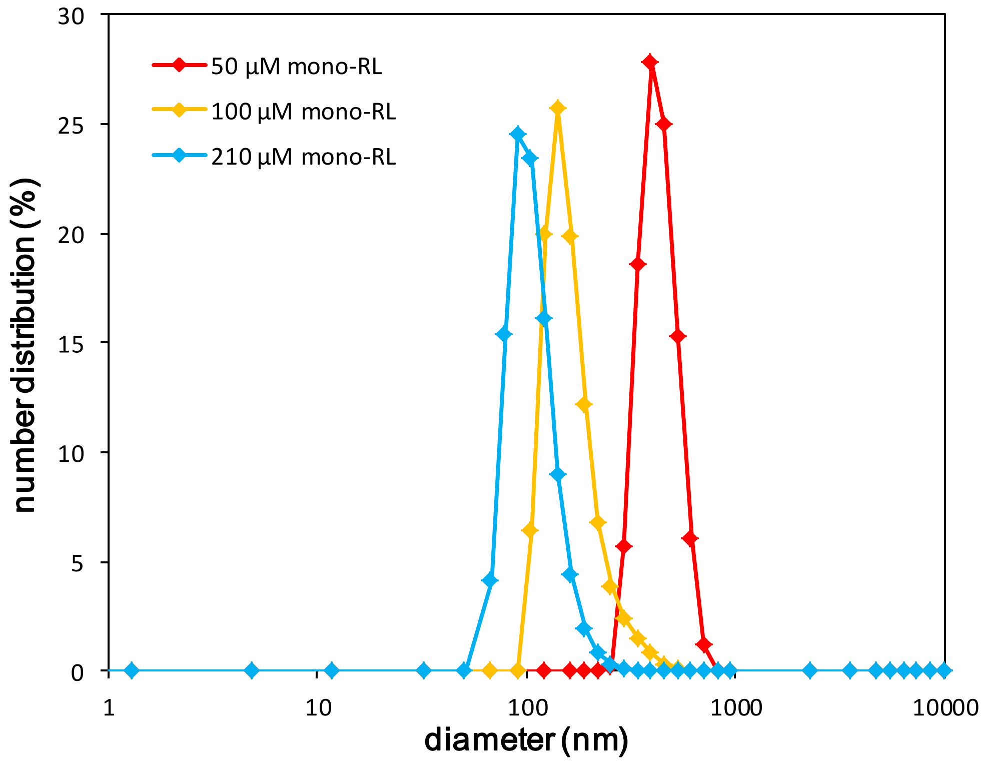

Figure S2. Typical number-based particle size distribution of the aggregate. Samples were obtained at the plateau of dodecane elution curves.

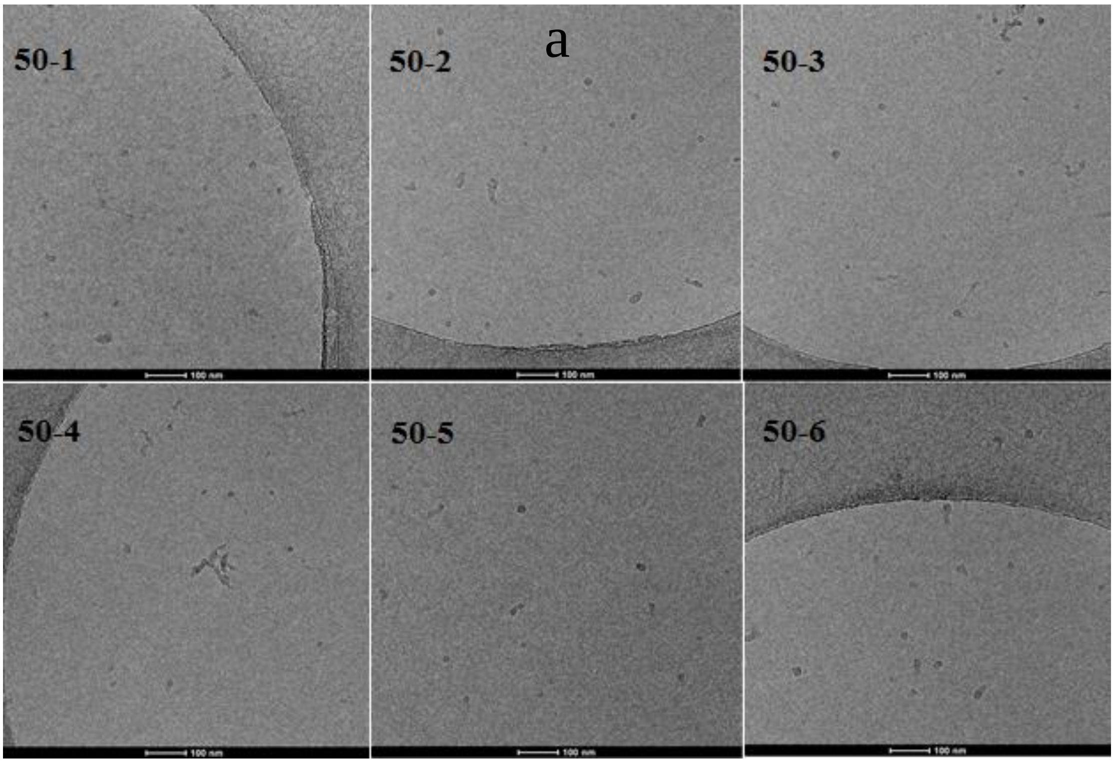
a
50-1
50-2
50-3
50-4
50-5
50-6

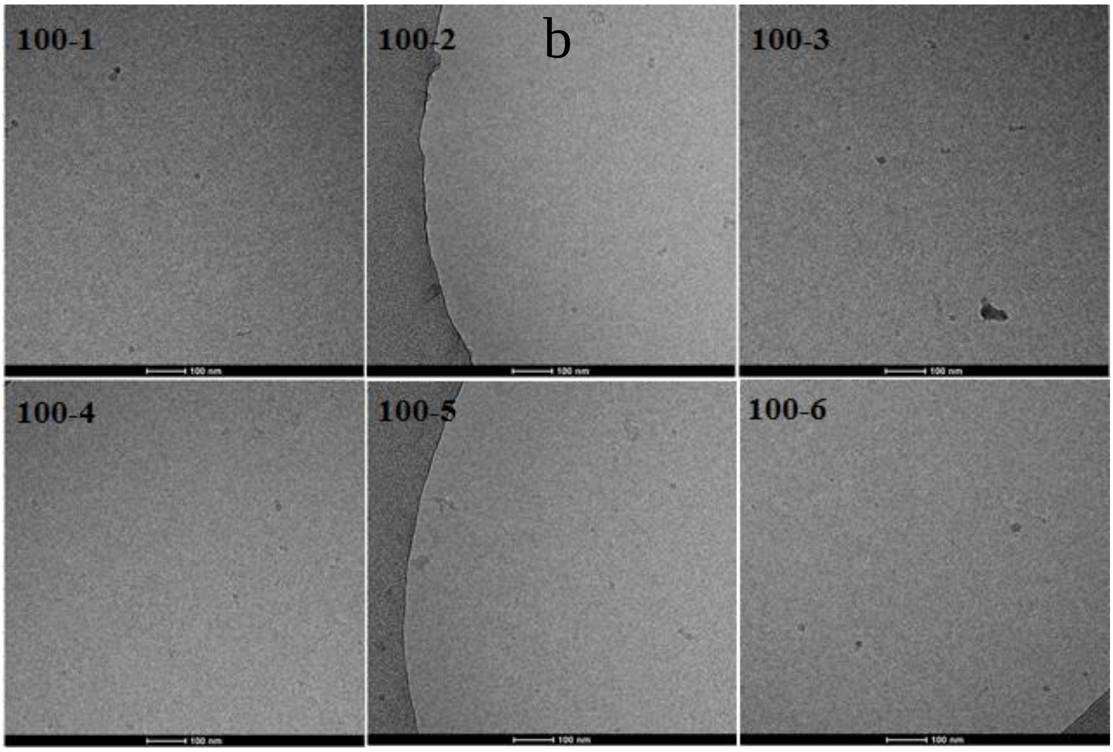
b
100-1
100-2
100-3
100-4
100-5
100-6

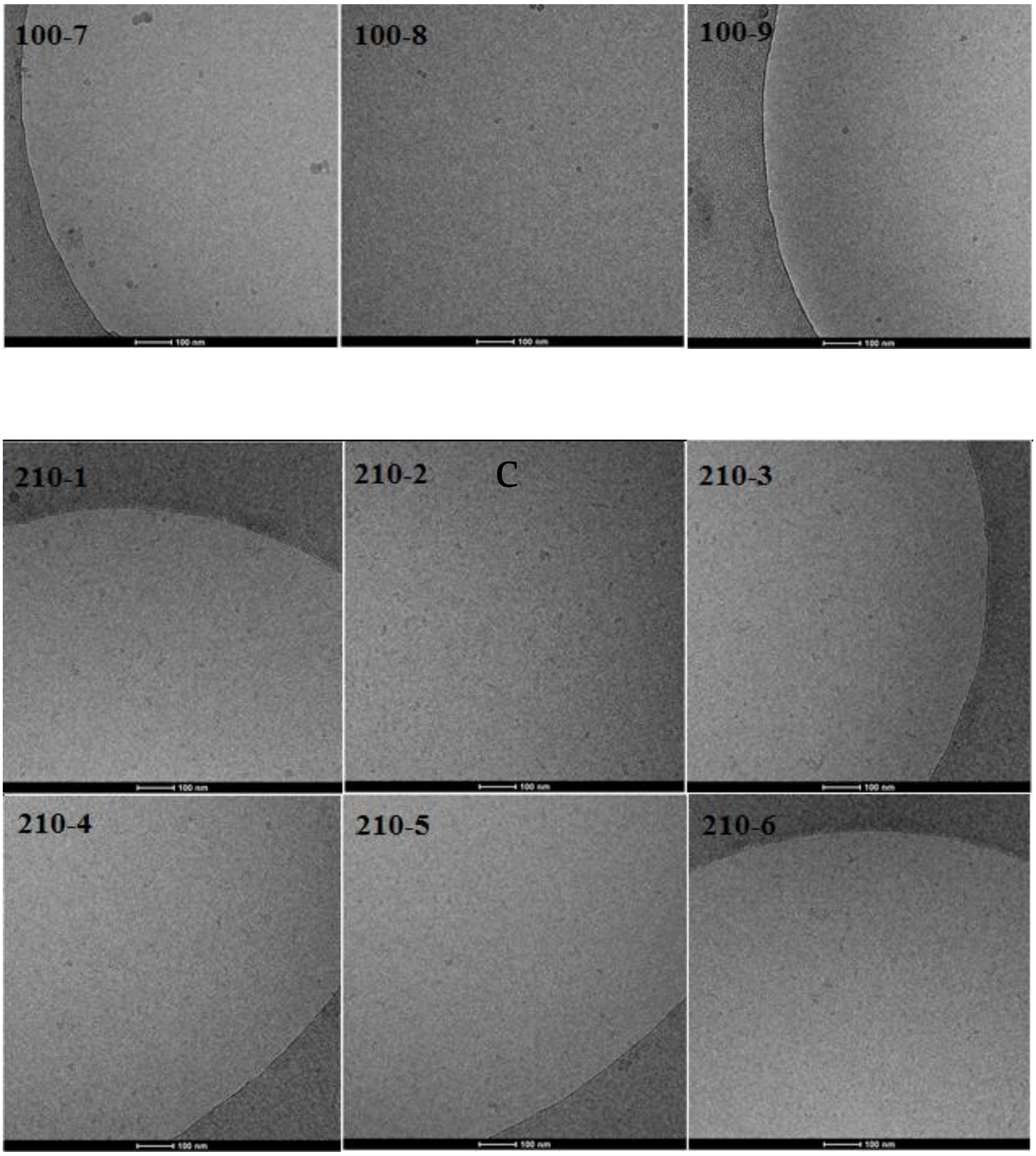

Figure S3 Cryo-TEM micrographs of dodecane-monoRL aggregates in effluent of flow cell in solubilization test for aggregate size calculation. Rhamnolipid concentrations are 50μM (a), 100μM (b), and 210μM (c).

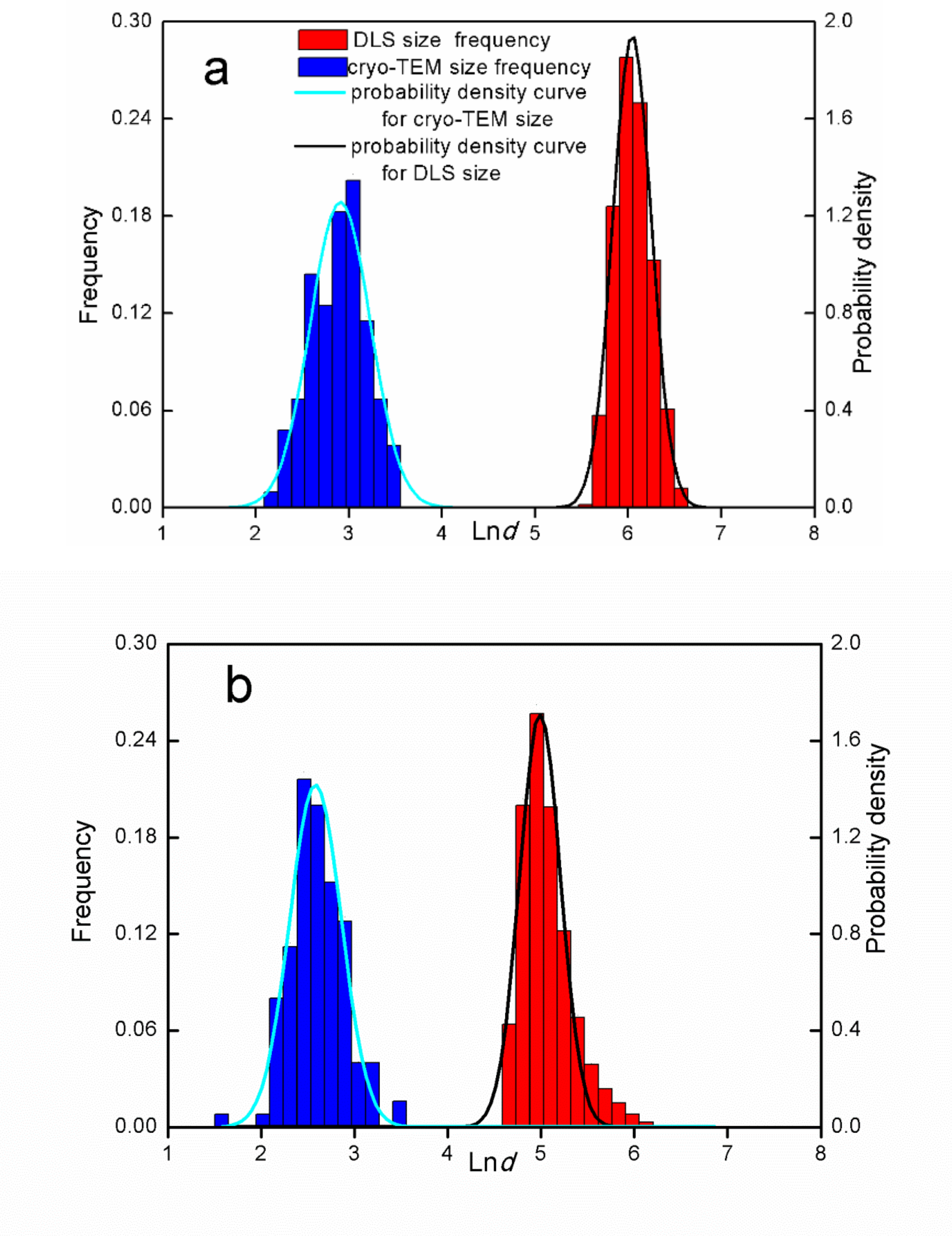
a
DLS size frequency
cryo-TEM size frequency
probability density curve for cryo-TEM size
probability density curve for DLS size
Frequency
Probability density
Lnd
b
Frequency
Probability density
Lnd

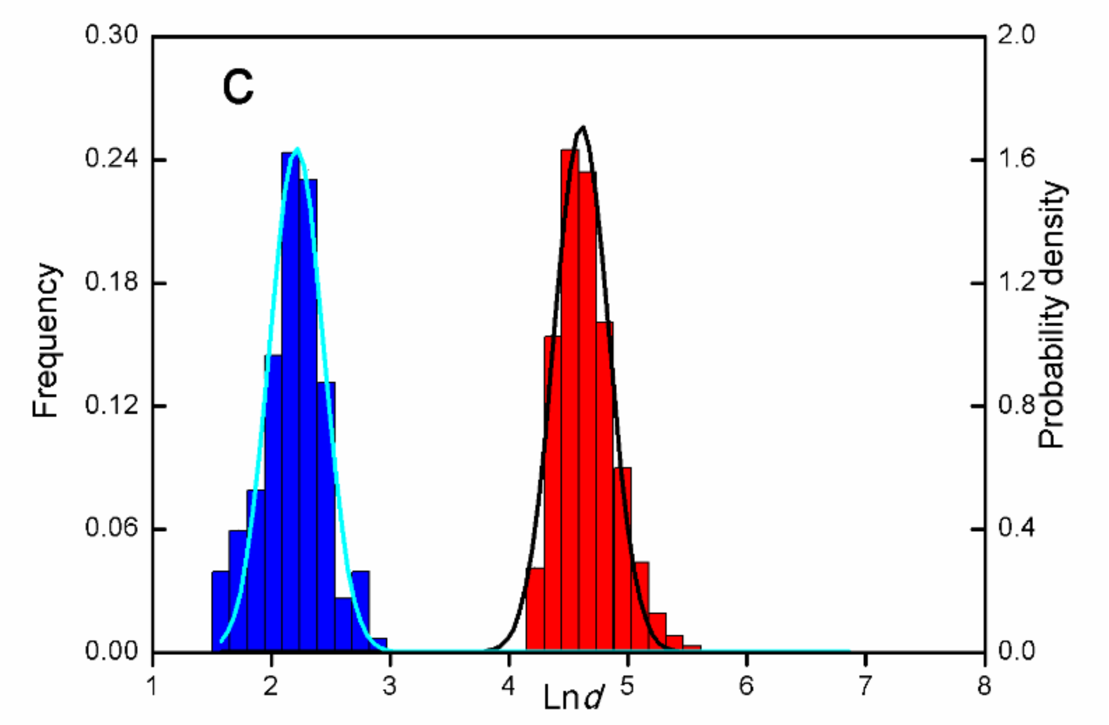

Figure S4 Gaussian regression for delineating dodecane-monoRL aggregate size distribution in the solublization experiment. The rhamnolipid concentrations in the influent are 50μM (a), 100μM (b), and 210μM (c).

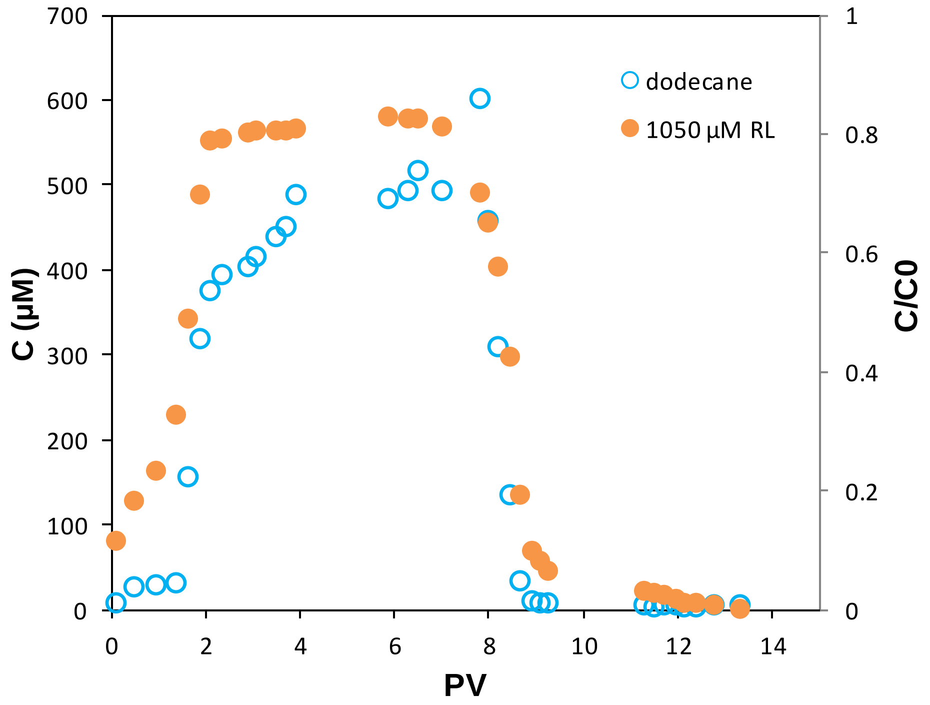

Figure S5 Elution curves of dodecane and Breakthrough curves of 1050 μM rhamnolipid during 1050 μM rhamnolipid elutiong the 2-D flow cell

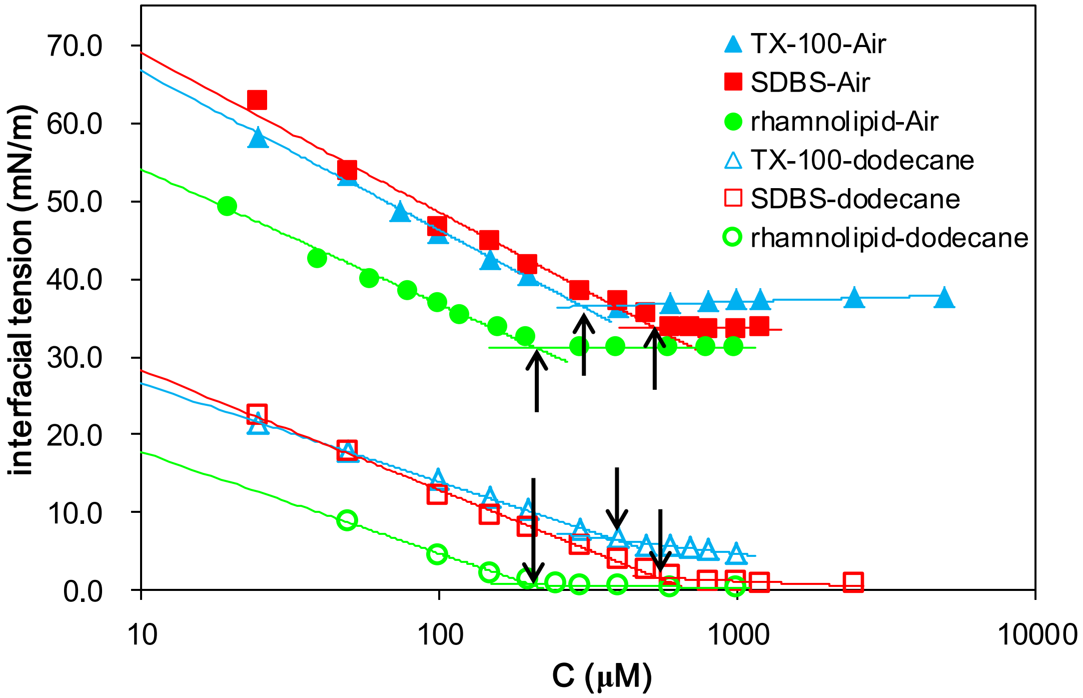

Figure S6 Interfacial tension versus surfactants concentration in AGW solution and CMCs of the surfactants

Methods of determing CMC

Surfactants (monoRL, SDBS, and TX-100) in AGW solution in a series of concentrations were prepared. The interfacial tensions between doecane and surfactants solution were measured at 30 ℃ using a tensiometer (JZ-200A, Chengde, China) based on the Du Noüy Ring method[1, 2, 3, 4]. The detailed procedures for the measurements were described by Zhong et al[1]. The CMCs were obtained by plotting the interfacial tension versus surfactants concentration. For reference purpose, surface tensions (interfacial tensions between air and solution) of surfactants solution were also measured.

The dependence of surface and interfacial tension on surfactants concentration in the AGW is presented in Fig. S6, SI. The CMCs of surfactants were calculated using

the method described by Zhong et al[5]. The CMCs of SDBS, Triton X-100, and monoRL obtained using the interfacial tension data are similar to those obtained using from surface tension data, indicating minimal impact of the type of non-aqueous phase on the CMCs. The CMC of the monoRL is significantly lower than that of SDBS and Triton X-100, showing stronger interfacial activity of this biosurfactant. The CMCs determined using interfacial tension data (584, 302 and 210 μM, respectively, for SDBS, TX-100 and the monoRL) were used for following experiments.

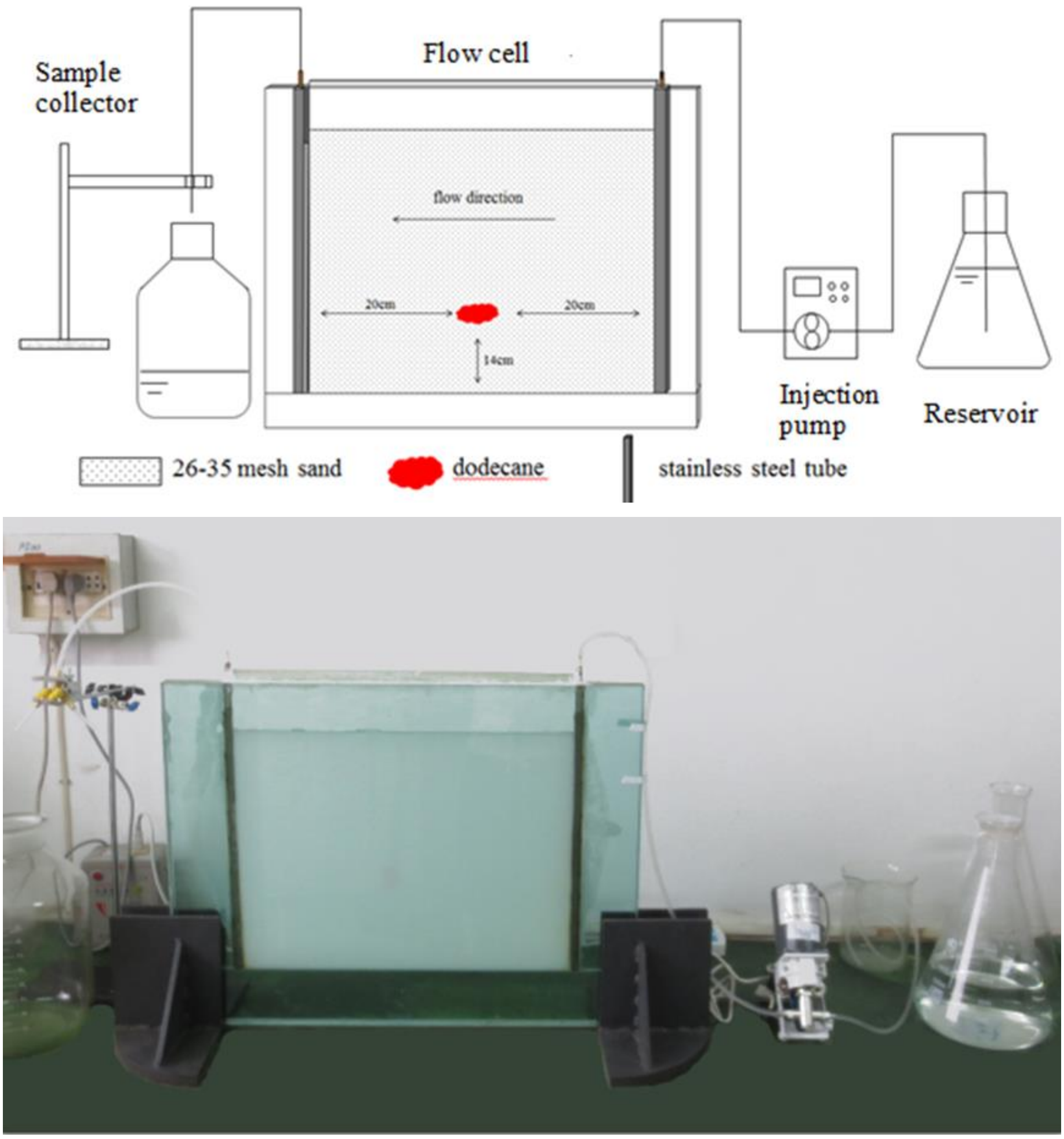

Figure S7 The diagram and image of the 2-D flow cell system.

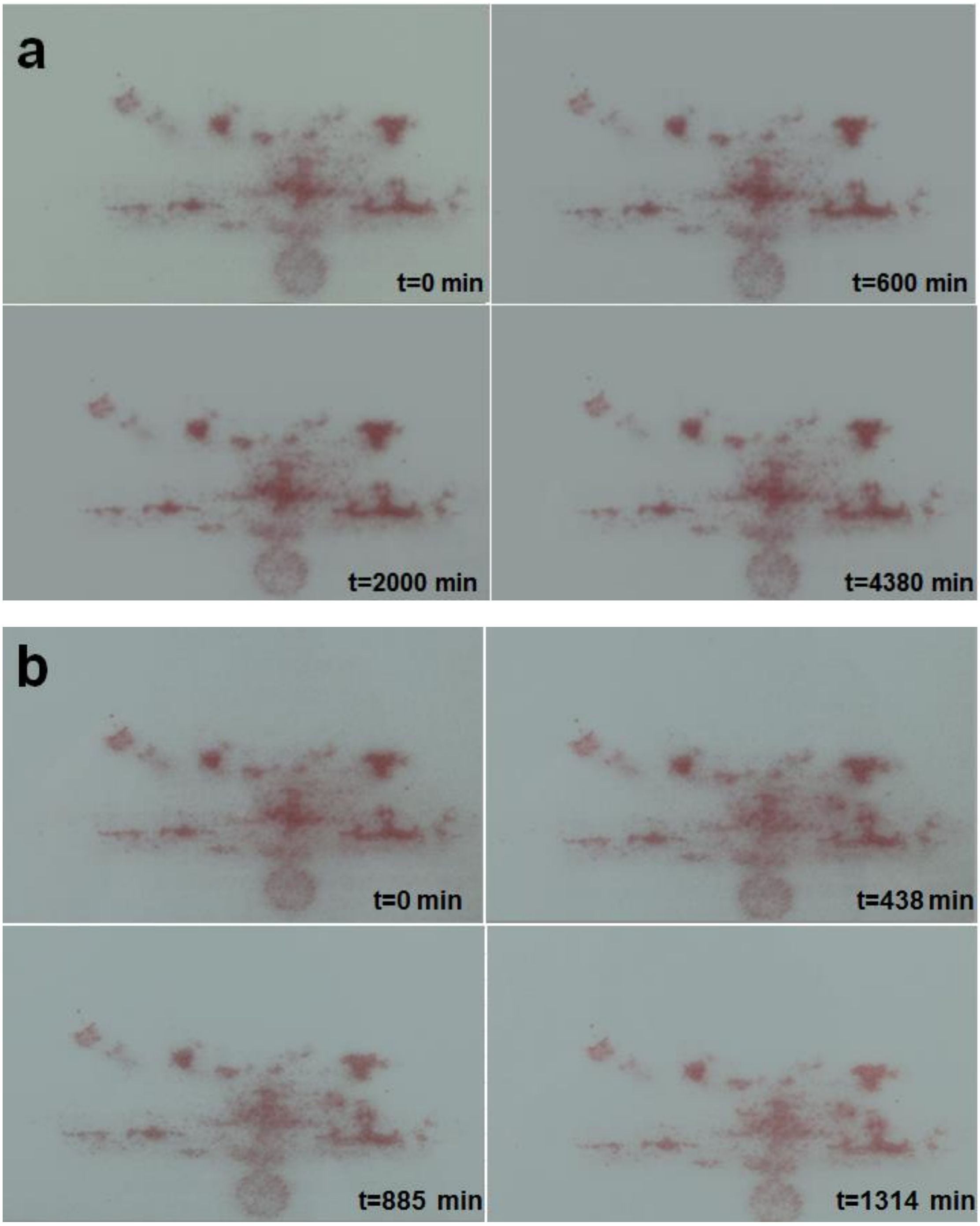
a
t=0 min
t=600 min
t=2000 min
t=4380 min
b
t=0 min
t=438 min
t=885 min
t=1314 min

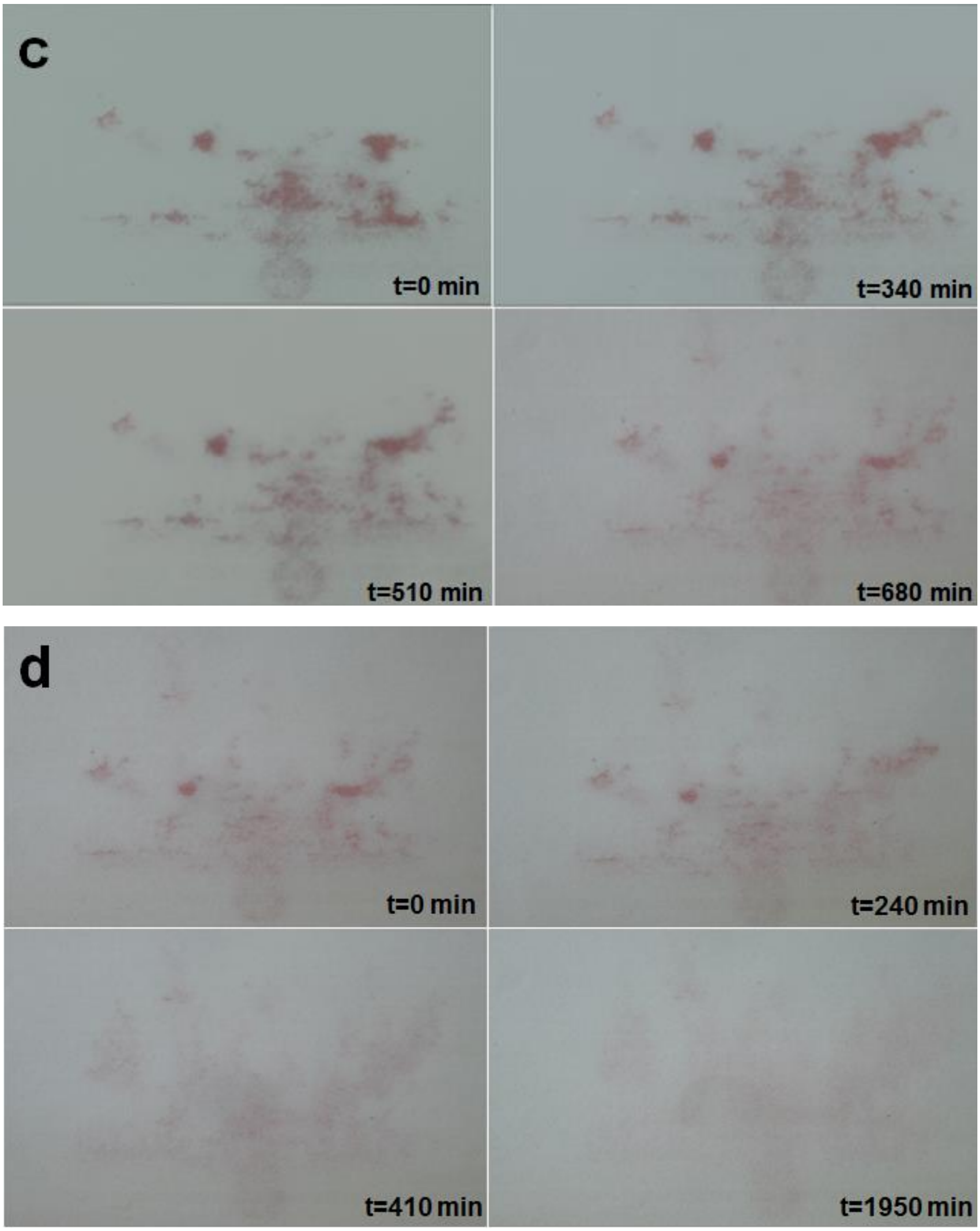

Figure S8 Dodecane phase configuration in the sand versus time with injection of AGW (a) and monoRL solution at concentrations of 50μM (b), 210μM (c), and 1050μM (d). The injection rate is 4 ml/min.

References

1. Zhong, H. *et al.* Aggregate-based sub-CMC solubilization of n-alkanes by monorhamnolipid biosurfactant. *New Journal of Chemistry* (2016).
2. Zhong, H. *et al.* Aggregate-based sub-CMC solubilization of hexadecane by surfactants. *RSC Advances***5**, 78142-78149(2015).
3. Yuan, X.; *et al.* Adsorption of surfactants on a Pseudomonas aeruginosa strain and the effect on cell surface lypohydrophilic property. *Applied microbiology and biotechnology***76**, 1189-1198(2007).
4. Zhong, H. *et al.* Aggregation of low-concentration dirhamnolipid biosurfactant in electrolyte solution. *RSC Advances* **5**, 88578-88582(2015).
5. Zhong, H. *et al.* Adsorption of monorhamnolipid and dirhamnolipid on two Pseudomonas aeruginosa strains and the effect on cell surface hydrophobicity. *Applied microbiology and biotechnology* **79**, 671-677(2008).
